# Genotypic distribution of human papillomavirus and phylogenetic analysis of E6 and E7 gene of HR-HPV variants isolated from Pakistani population

**DOI:** 10.1097/MD.0000000000032651

**Published:** 2023-01-13

**Authors:** Sameen Ahmed, Ayesha Vajeeha, Muhammad Idrees, Abrar Hussain, Rakhtasha Munir, Gulshan Zaidi, Khadija Zahid, Rizwan Ahmed, Zareen Fatima, Shazia Rafique, Niaz M. Achakzai

**Affiliations:** a Centre of Applied Molecular Biology (CAMB), University of the Punjab, Lahore, Pakistan; b University of Peshawar, Peshawar, Pakistan; c Department of Biotechnology, BUITEMS, Quetta, Pakistan; d Centre of Excellence in Molecular Biology (CEMB), University of the Punjab, Lahore, Pakistan; e Government College University Faisalabad (GCUF), Faisalabad, Pakistan; f Department of Molecular Biology, DNA Section, Legal Medicine Directorate, Ministry of Public Health, Kabul, Afghanistan.

**Keywords:** cervical cancer, human papillomavirus, Pakistani population, phylogenetic analysis

## Abstract

High-risk-human papillomavirus (HR-HPV)-induced cervical cancer is the second most common cause of death among females worldwide. HPV16 is the most prevalent HR-HPV infection worldwide. This study found the genotypic distribution of HR-HPV in the local population and investigated the sequence variations among the E6 and E7 oncogenes of the local HPV16 genotype to the E6 and E7 oncogenes of the foreign HPV16 genotypes and constructed a phylogenetic relationship based on nucleotide sequence comparison among the variants identified in our study along with previously reported isolates that were obtained from different regions of the world. The samples were collected from patients with cervical cancer. Genomic DNA was extracted, and HR-HPV genotypes were determined using real-time PCR. The HPV16 E6 and E7 genes were amplified and sequenced. A HPV16 phylogenetic tree was constructed using the maximum likelihood method with MEGA 7. HPV16 was the most prevalent human papillomavirus (HPV) type identified in the present study. HPV16 isolates belonged to the A1 sublineage of the European branch. Twenty-one nucleotide sequences were included in this analysis. The first, second, and third codon positions are also included. The final dataset included 776 positions.

## 1. Introduction

Cervical cancer is the fourth most common cancer among women globally, accounting for 8% of women’s deaths annually.^[1]^ Cervical cancer proceeds in four steps. The first step is metastatic epithelial tissue infection at the cervical transformation region, the second is a persistent viral infection, the third is the development of cervical precancer by persistently infected epithelium, and the last is invasion along the epithelial basement membrane.^[2]^

Papillomaviruses are circular DNA viruses that are small, double-stranded, non-enveloped, approximately 8 Kb in size, and have a diameter of approximately 55 nm.^[3–5]^ Human papillomaviruses (HPVs) contribute to several diseases, ranging from benign warts to invasive cervical cancers. HPV has been confirmed as an etiological agent of cancers of the urogenital and oropharyngeal regions. More than 200 HPV types have been thoroughly characterized.^[4–6]^ Among these HPV genotypes, only 13 (16, 18, 31, 33, 35, 39, 45, 51, 52, 56, 58, 59, and 66) are considered high-risk-human papillomavirus (HR-HPV) types by the International Agency for Research on Cancer (IARC).^[7]^

The HPV genome contains 3 distinct regions. The first region is an early region that constitutes 50% of the genome and includes E1, E2, E4, E5, E6, and E7. The second region within the genome, known as the late region (L), comprises 40% of the genome and includes L1 and L2. The third region, which represents the remaining 10% of the genome, is the regulatory region.^[8]^

The host clears most HPV infections within two years and persistent infection with HR-HPV causes cancer. Integration of the viral genome into the host genome causes persistent oncogenic expression.^[9,10]^ The viral oncoproteins E6 and E7 of HR-HPV play major roles in cervical cancer.^[3,4]^ The E6 protein stimulates degradation of the tumor suppressor p53, resulting in the creation of a trimeric complex of p53, E6, and E6-AP, which promotes cell proliferation. E6-stimulated degradation of p53 increases tumor cell proliferation.^[11]^ E7 contains three conserved domains, namely, CR1, CR2, and CR3. The CR2 domain contains an LXCXE motif that binds to retinoblastoma protein and related proteins p107 and p130. Because of this binding, retinoblastoma dissociates from transcription factor E2F. The release of E2F causes the cell cycle to enter the S phase prematurely.^[12,13]^

HPV 16/18 cause for 70% of all cervical carcinomas. However, the actual burden of cervical cancer on the Pakistani population remains unclear. Among patients with cervical carcinoma, the HPV positivity rate varies from 18%^[14]^ to 98.33%.^[15,16]^ The reason for this is poor documentation of the screening, vaccination, and epidemiology of the disease.^[17]^ In Pakistan, it is the third most common cancer among the female population. At any given time, approximately 0.5% of women harbor HPV 16/18, and 88.1% of highly spreadable cervical cancer cases are due to HPV 16/18.^[18]^ Further data on the safety and efficacy of HPV vaccines in Asia and Pakistan are required.

The human papillomavirus 16 (HPV16) intratypic variants were further divided into four lineages: A, B, C, and D, depending on the geographical origin of the population from which they were isolated. A was classified into three European sublineages, (A1, A2, and A3), and one Asian A4 sublineage. Lineage B was divided into two African sublineages, B1 and B2, whereas lineage C included the African sequences. Lineage D consists of the D1, D2, and D3 sublineages, which contain North American and Asian American sequences.^[19]^

The most important aim of this study was to evaluate the sequence variations among the E6 and E7 oncogenes of HPV16 via a phylogenetic analysis of the sequences identified in our study with those of the sequences from the different regions of the world reported previously and to determine the phylogenetic relationship among Pakistani sequence variants, and the worldwide reported sequence variants.

## 2. Methods

### 2.1. Sample collection

A total of 132 cervical swabs were collected from women with cervical cancer across Punjab, Pakistan aged 25 to 72 years enrolled at the Institute of Nuclear Medicine and Oncology Lahore, Pakistan. The swabs were immediately transferred to tubes containing 500 µL phosphate buffer saline. The samples were further processed at the Virology Laboratory at the Center of Excellence in Molecular Biology in Lahore, Pakistan.

Samples were obtained from patients willing to provide informed consent and from those who had not received cervical cancer treatment in the previous year. Women who were hysterectomized, pregnant, or infected with other viruses were excluded from the study.

### 2.2. DNA extraction and genotype detection

Ethanol precipitation was used to extract the genomic DNA. HR-HPV types were determined by real-time PCR using the HPV 14 Types Detection Kit (Healgen Scientific

Houston, Texas). It can evaluate 14 types of HPV (16, 18, 31, 33, 35, 39, 45, 51, 52, 56, 58, 59, 66, and 68). Samples that were positive for HPV16 were selected for sequence analysis.

### 2.3. PCR amplification and DNA sequencing

HPV E6 and E7 genes were amplified using coding region primers for HPV E6 and E7 (Table 1). The two PCR reactions were conducted separately in 20 µL volumes containing 10 pmol forward and reverse primers, 10 mM dNTPs, 100 ng genomic DNA, 2.5 mM MgCl_2_, 1 U of Taq DNA polymerase and 10× PCR buffer. In the BioRad T100^TM^ thermocycler, 35 amplification cycles were run with a 95°C denaturation step for 45 seconds, annealing (both for E6 and E7) for 30 seconds at 58°C, and extension for 30 seconds at 72°C, with an initial denaturation of 5 and 10 minutes of final extension. Agarose gel electrophoresis was used to analyze the PCR products.

**Table 1:** Primer pairs designed for the amplification of HPV16 E6 and E7 genes.

Primer name	Primer sequence	Product size	Annealing temperature
HPV-16 E6 F	5′-ATGCACCAAAAGAGAACTGC-3′	477 bp	58°C
HPV-16 E6 R	5′-TTACAGCTGGGTTTCTCTACG-3**′**		
HPV-16 E7 F	5′-ATGCATGGAGATACACCTAC-3**′**	297 bp	58°C
HPV-16 E7 R	5′-TTATGGTTTCTGAGAACAGATGGG-3**′**		

HPV16 = human papillomavirus 16.

The PCR products were purified using the QIAquick PCR Purification Kit (Qiagen

Germantown, Maryland) (28104). The amplified products were further sequenced using the BigDye Terminator Sequencing Kit (Applied Biosystems, Waltham, Massachusetts, 4337455), and the sequencing reaction was performed in both directions using an ABI PRISM 3100 Genetic Analyzer (Applied Biosystems). The sequences were analyzed using BLAST.

### 2.4. Phylogenetic tree generation

A HPV16 phylogenetic tree was constructed using the nucleotide sequences of the E6 and E7 obtained in this study. Reference sequences for the eight sublineages were obtained from GenBank (www.ncbi.nlm.nih.gov) (Table 2). The CLUSTALW program was used for pairwise and multiple sequence alignment. The evolutionary history was inferred using the Maximum Likelihood method based on the general time reversible model using the MEGA7 version.^[20,21]^

**Table 2:** HPV16 sub-lineages GenBank accession numbers.

A1	A2	A3	A4	B1	B2	C	D3
K02718	AF536179	HQ644236	HQ644234	AF472508	HQ644298	AF472509	HQ644289
KU298880	KU053892		AF534061	HQ644296		AB818690	AF402678
	FJ610152		HQ644261	AF536180			HQ644285

HPV16 = human papillomavirus 16.

## 3. Results

Of 132 women with cervical cancer, 72 tested positive for HR-HPV. The most prevalent HR-HPV type was HPV16. The genotypic distributions of HPV in this study are shown in Table 3.

**Table 3:** Genotypic distribution of HR-HPV in the cervical cancer patients of the Pakistani population.

Total no of patients	132
Patients positive for HPV	72 (54.5%)
Patients positive for HPV16	61 (46%)
Patients positive for HPV18	1 (0.8%)
Patients positive for other HR-HPV	3 (2.3%)
Patients with coinfection of HPV16 and HPV18	2 (1.5%)
Patients with a coinfection of HPV16 and other HR-HPV	4 (3%)
Patients with a coinfection of HPV16, HPV18 and other HR-HPV	1 (0.8%)

HPV16 = human papillomavirus 16, HR-HPVs = high-risk human papillomavirus.

### 3.1. PCR amplification and sequencing

**Figure 1.**
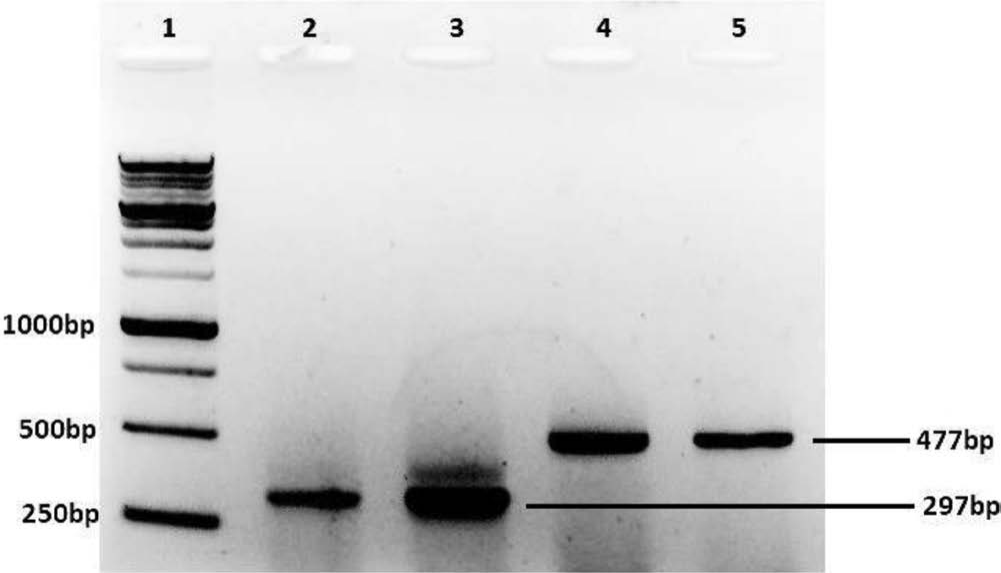
Amplification of HPV16 E7 and E6 Genes, Lane 1: 1 kb DNA ladder (Thermo Scientific # SM1163); Lane 2 to 3: E7 Gene (297 bp), Lane 4 to 5: E6 Gene (477 bp). HPV16 = human papillomavirus 16.

The E6 and E7 genes of HPV16 were amplified using gene-specific primers. The product sizes of E7 and E6 were 297 and 477 bp, respectively, as shown in Figure 1. Sequences of the amplified products were confirmed by sequencing the reaction in duplicate.

### 3.2. Phylogenetic analysis

**Figure 2.**
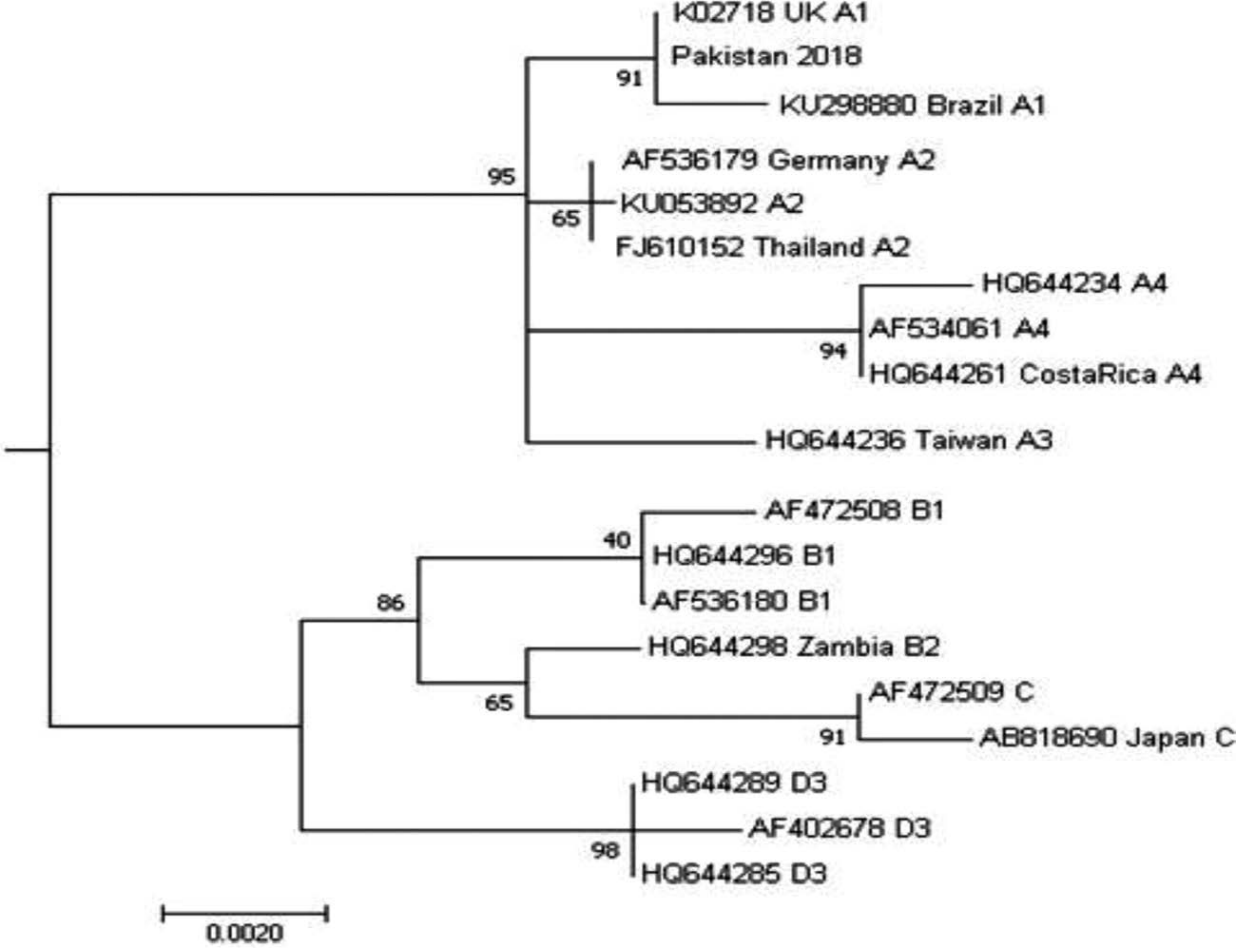
Phylogenetic tree of E6 and E7 genes of different HPV16 isolates. HPV16 = human papillomavirus 16.

Phylogenetic analysis based on HPV16 E6 and E7 gene sequences showed that the Pakistani isolate in this study lies in the A1 sublineage of the A lineage of the alpha-papillomavirus genus alpha-9 (Fig. 2). Twenty-one nucleotide sequences were used for analysis. The first, second, and third codon positions are also included. The final data set included 776 positions.

## 4. Discussion

Human papillomavirus (HPV) belongs to the *Papillomaviridae* family. It comprises of more than 200 types isolated from humans.^[4–6]^ The HPV genome comprises double-stranded DNA that is 8 kb in size and consists of eight genes: E1, E2, E4, E5, E6, E7, L1, and L2.^[2–5,8]^ To initiate and maintain HPV-associated malignancies, oncoproteins E6 and E7 are required and are expressed in transformed cells.^[22]^

The identification of HPV variants is essential for developing diagnostic tests and designing vaccines and therapeutics.^[23]^ Previous studies have reported that the distribution of HPV variants is related to geographic or racial distribution.^[24,25]^ The most crucial purpose of this study was to represent the division of different HPV16 lineages among the Pakistani population, according to the classification of Yamada et al^[26]^

Cervix uteri is the fourth most common cancer in women worldwide.^[1]^ In 2018, 311,365 deaths and 569,847 new cases were reported globally. Squamous cell carcinoma cases followed by adenocarcinomas have been reported in most cases.^[27]^ Estimates from 2012 showed a 7.9% prevalence rate of HPV-related cancers in Pakistan, whereas other cancers (anogenital and head/neck cancers) collectively accounted for 0.5%.^[28]^ According to the ICO/IARC HPV Information Center, approximately 5008 new cervical cancer cases and 3197 new deaths were reported in Pakistan in 2020. In Pakistani women, cervical cancer is the third most common cause of cancer and death due to cancer and the second most common cancer between the ages of 15 and 44 years.^[18]^

In addition to HPV, other factors also cause cervix uteri cancer. These include tobacco smoking, specific nutritional deficits, Co-infection with Chlamydia trachomatis, long-term hormonal contraceptive use, high parity, co-infection with human immunodeficiency virus, immunosuppression, and herpes simplex virus type-2. In addition to viral factors such as different virus types, viral integration, and viral load, both immunological and genetic host factors can be critical in causing cancer.^[29]^ Data from Pakistan show that 1.7% of women use hormonal contraception (pill, injectable, or implant), the total fertility rate (live births per woman) is 3.4%, and smoking is prevalent in 2.6% of women with HPV-related cancer.^[18]^

This study found that 54.5% of the patients with cervical cancer were positive for HPV. The most prevalent type was HPV16 (46%). Only 0.8% of the women were positive for HPV18, and 2.3% had other HR-HPV infections. A total of 1.5% of women were found to have co-infection with types 16 and 18. 3% of the women were co-infected with HPV16 and other HR-HPVs, whereas only 0.8% were co-infected with type 16, 18, and other HR-HPVs. In previous studies on cervical cancer patients, the prevalence rates of HPV were reported to be 88% in cancer patients and 2.8% in non-cancer patients,^[14]^ 94.81%,^[30]^ 88.0%,^[17]^ 87.5%,^[31]^ and 2% in non-cancer patients.^[32]^ Aziz et al^[33]^ reported high-risk types of HPV16 (4.16%), HPV33 (8.33%), HPV45 (12.5%), and HPV18 (6.25%) in Pakistan patients. In another study from Punjab, Pakistan, the HR-HPV prevalence was reported to be 57%, while HPV16, 18, and 45 were 18%, 6%, and 1%, respectively.^[34]^

The collected samples were categorized into the A1 sublineage of the alpha 9 species of HPV16. To the best of our knowledge, this is the first report to classify HPV isolated from Pakistan at the sublineage level. The Pakistani HPV16 isolate fell within isolates from the UK (reference sequence K02718 for the A1 sublineage) and Brazil, with 99% homology among the sequences. A robust bootstrap value of 91% supports this hypothesis. Previously, the most detailed study on HPV categorization in Pakistan classified HPV16 into the alpha 9 species group.^[32]^ Very few small-scale studies have reported the incidence of HPV16 and HPV18 infections in patients with or without cancer.

The HPV16 E6 and E7 genes and the LCR region were analyzed in Uruguayan women. The E6 and E7 nucleotide sequences presented 18 non-synonymous mutations. These sequences belonged to the European lineage. Two belonged to the African lineage and three were from the Asian American and North American lineages.^[19]^ A study from Jeddah, Saudia Arabia based on phylogenetic analysis of HPV16 L1 and E6 nucleotide sequences showed the distribution of HPV16 in six lineages A1, A2, A4, B1, C, and D2.^[35]^

As the sexual transmission remains the main route of HPV infection, sociocultural barriers interfere with the true prevalence of HPV in Pakistan. Owing to a lack of awareness, few females opt for HPV screening, and HPV infection remains unnoticed unless it progresses to a severe problem. Phylogenies are essential to map the groups in which HPV isolates lie because they can be used to identify the region that can be the probable cause of the spread of HPV in any country.

The limitation of this study was the small number of available samples. Samples from different regions of Pakistan, except for Punjab, could belong to other lineages or sublineages of HPV16.

## 5. Conclusion

The HPV16 isolates from Pakistan belong to the European lineage. These data will help better understand the molecular epidemiology of HPV. This will also help to develop therapeutic vaccines and molecular diagnostic tools for the Pakistani population.

## Acknowledgments

We are grateful to the healthcare professionals of INMOL Hospital, Lahore, for helping collect clinical data and samples from cervical cancer patients.

## Author contributions

SR, AH, and MI planned this study. SA, AV, RM, GZ, and KZ contributed to laboratory work, and RA helped with sample collection and laboratory work. ZF helped with data analysis; SA, AV, and NMA contributed to the writing and editing of the manuscript. All the authors have read and approved the final manuscript. All the authors critically reviewed the manuscript and approved the final draft.

**Conceptualization:** Muhammad Idrees, Abrar Hussain, Shazia Rafique.

**Formal analysis:** Gulshan Zaidi, Khadija Zahid, Zareen Fatima.

**Investigation:** Sameen Ahmed, Rakhtasha Munir, Ayesha Vajeeha.

**Methodology:** Sameen Ahmed, Ayesha Vajeeha, Rakhtasha Munir, Gulshan Zaidi, Rizwan Ahmed.

**Supervision:** Shazia Rafique.

**Visualization:** Khadija Zahid.

**Writing – original draft:** Abrar Hussain, Shazia Rafique.

**Writing – review & editing:** Muhammad Idrees, Abrar Hussain, Niaz M. Achakzai.
